# Stiff skin syndrome: long-term follow-up^☆^

**DOI:** 10.1016/j.abd.2023.07.013

**Published:** 2024-04-24

**Authors:** Jessica Lana Conceição e Silva Baka, Tauana Ogata Coelho da Rocha, Marcella Soares Pincelli, Luciana Paula Samorano, Maria Cecília da Matta Rivitti-Machado, Zilda Najjar Prado de Oliveira

**Affiliations:** a Pediatric Dermatology, Hospital das Clínicas, Faculty of Medicine, Universidade de São Paulo, São Paulo, SP, Brazil; b Dermatopathology, Hospital das Clínicas, Faculty of Medicine, Universidade de São Paulo, São Paulo, SP, Brazil

Dear Editor,

Stiff skin syndrome (SSS) is a rare, fibrosing, non-inflammatory, and progressive disease that manifests itself at birth or in early childhood. The dermatological picture is characterized by sclerotic plaques, with a hard consistency, with or without hypertrichosis. The lesions occur predominantly in the limbs and scapular and pelvic girdles, causing joint deformities and gait and postural changes.^1^, ^2^, ^3^ Characteristically, there are no systemic symptoms, such as Raynaud’s phenomenon, periungual changes, and visceral involvement, which helps to differentiate it from systemic sclerosis, localized cutaneous sclerosis, collagenomas, neonatal sclerema, scleromyxedema and mucopolysaccharidoses.^4^, ^5^

The diagnosis is based on clinical and histopathological findings. The former include hereditary (fibrillin-1 gene) or sporadic condition (mutation in the transforming growth factor-beta binding protein 4), prominent skin thickening in areas with abundant fascia (buttocks and thighs), joint mobility limitation, hypertrichosis and absence of systemic changes. The microscopic findings are: thickening of collagen with deposition of mucin; fascial sclerosis; absence of significant inflammation; horizontal orientation of thickened collagen fibers and adipocyte entrapment.^2^, ^6^, ^7^, ^8^, ^9^, ^10^, ^11^

The treatment is challenging and there are no established guidelines for patient care. Therefore, most of the reported therapies are empirical, such as methotrexate, topical and systemic corticosteroids, UVB-NB phototherapy and immunoglobulin, without satisfactory results.^9^ Non-drug therapies, such as motor physical therapy, have been shown to be important in preventing muscle contractures and postural sequelae.^4^, ^5^

Five patients with SSS were followed at the Pediatric Dermatology outpatient clinic of Hospital das Clínicas, Faculty of Medicine, Universidade de São Paulo, aged 11 to 19 years, for an average period of seven years (Table 1). All had segmental hardened subcutaneous plaques (Fig. 1), with a cobblestone pattern (Fig. 2). All cases showed a difference in length between the limbs, and two had postural changes and pain during physical activity. Family history of SSS or consanguinity of the parents was not observed.

**Table 1:** Clinical and histopathological findings of patients with SSS.

	Case 1	Case 2	Case 3	Case 4	Case 5
**Gender**	M	F	F	M	F
**Location**	Middle to lower back and hips on the L	Lateral of R breast, to R thigh, flank and back on the L	Proximal anterior aspect of L thigh, abdomen, ipsilateral gluteal region	R shoulder, lateral upper back, proximal portion of RUL	Proximal anterior aspect of R thigh, ipsilateral abdomen
**Age at diagnosis**	9 years	9 years	6 years	5 years	5 years
**Current age**	19 years	19 years	12 years	13 years	11 years
**Thickening of collagen fibers and deposition of mucin**	Yes	Yes	No	Yes	Yes
**Fascial sclerosis**	Yes	No fascia represented	No fascia represented	No fascia represented	No fascia represented
**Absence of inflammation**	Yes	Yes	Yes	Yes	Yes
**Horizontal orientation of thickened collagen fibers**	Yes	Yes	No	Yes	Yes
**Adipocyte entrapment**	Yes	Yes	No	Yes	Yes
**Time of follow-up**	7 years	11 years	4 years	6 years	6 years
**Drug treatment/phototherapy**	Methotrexate 15 mg per week (38 months), prednisone 30 mg per day (5 months)	Methotrexate 15 mg per week (15 months), NB-UVB (12 months)	‒	‒	‒
**Physical therapy and regular exercise**	Yes	Yes	No (non-adherent patient)	Yes	Yes
**Limbs of different length**	Yes	Yes	Yes	Yes	Yes
**Postural change**	Yes	Yes	Yes	Yes	Yes
**Pain in the affected limb during physical activity**	No	No	Yes	No	Yes
**Disease stability**	Yes	No	No	Yes	No

R, Right; L, Left; RUL, Right Upper Limb; F, Female; M, Male.

**Figure 1:**
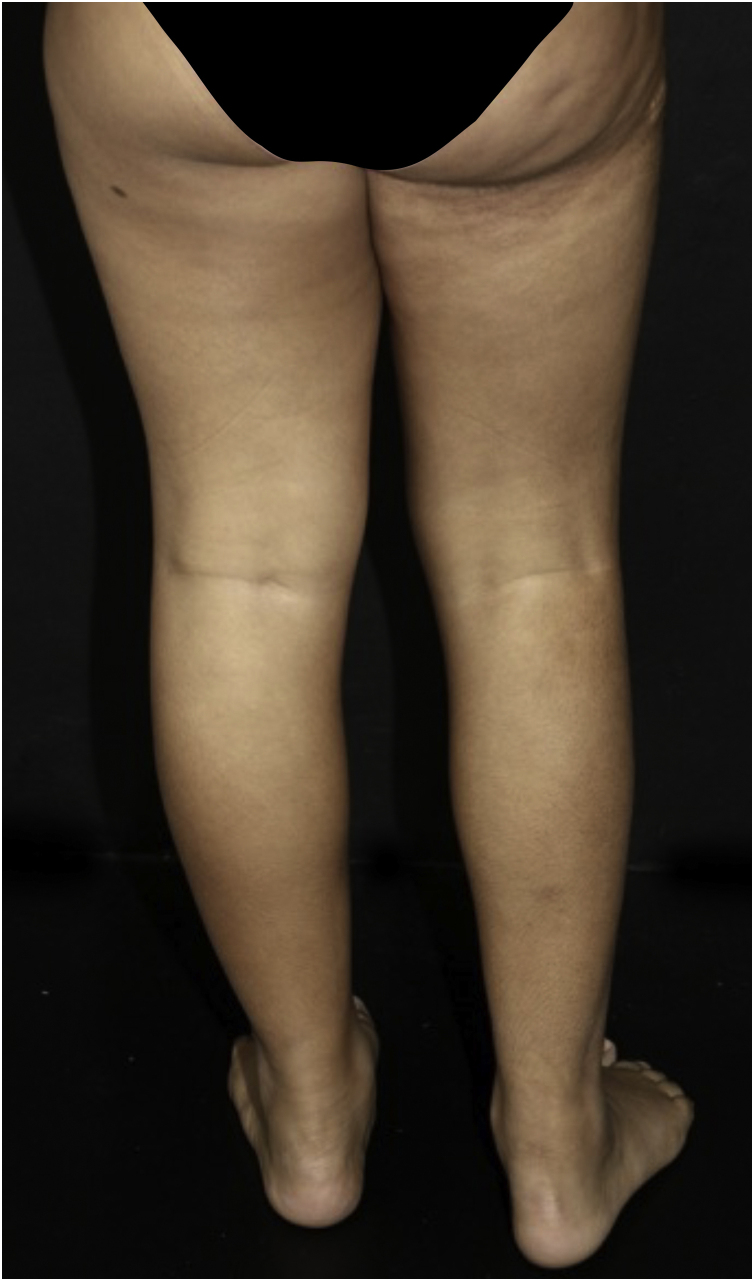
Stiff skin syndrome. An 11-years-old female patient, with sclerotic plaques showing a cobblestone pattern on the right gluteal and thigh region, also with a difference in length between the limbs.

**Figure 2:**
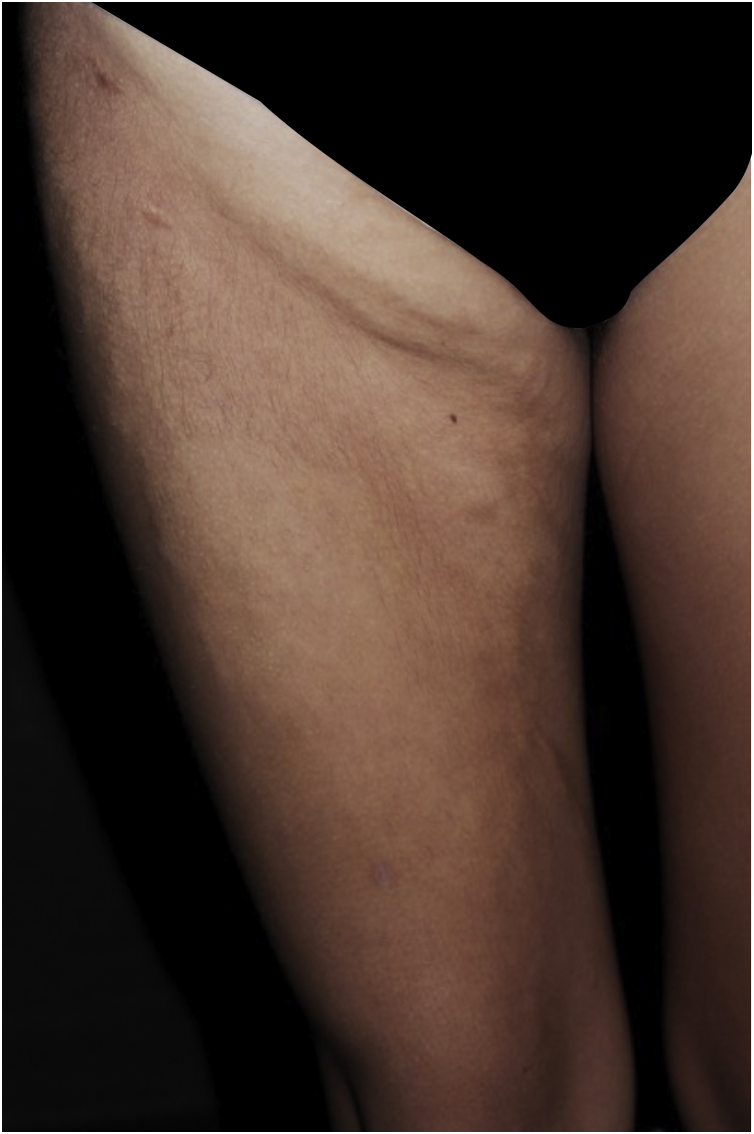
Stiff skin syndrome. An 19 years-old female patient, with sclerotic plaques showing a cobblestone pattern on the thigh and right inguinal region.

Patients underwent skin biopsy for histopathological examination (Fig. 3). Two patients underwent magnetic resonance imaging (MRI) aiming to better assess the extent of the lesion. MRI revealed slight skin thickening and vascular ectasia in the subcutaneous plane adjacent to the site of involvement.

**Figure 3:**
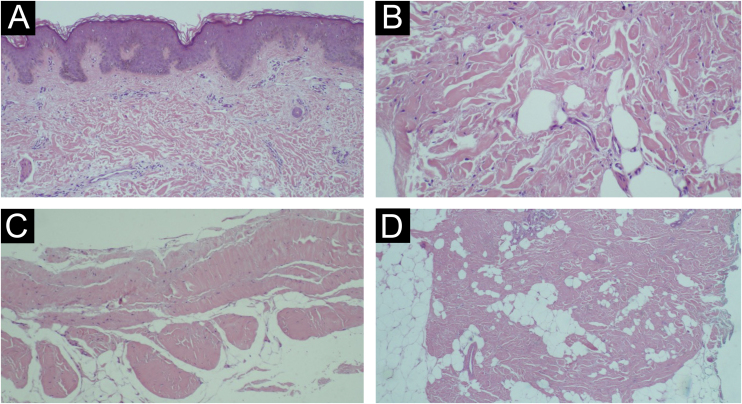
Histopathology. (A) Absence of significant inflammation; (B) Thickening and horizontalization of collagen fibers and mild dermal mucinosis; (C) Sclerosis and densification of the fascia; (D) Adipocyte entrapment and thickening of the septa (Hematoxylin & eosin staining).

The set of findings confirmed the diagnosis of SSS. Two patients were prescribed methotrexate and phototherapy with UVB-NB, which were later withdrawn due to lack of response. Treatment with physical therapy and regular physical exercise was recommended to all. At the last evaluation, stability or slow progression of the condition was observed (Table 1).

SSS is a challenging disease in clinical practice, given its rarity, scarcity of reported cases, and the slowly progressive nature of the disease, leading to late diagnosis.^1^, ^2^, ^3^

Deng et al.^3^ reported predominance in female patients, and the same was observed in this case series. Uni- or bilateral involvement and difference in length between the limbs were observed in all patients, findings in agreement with data in the literature.^1^, ^2^, ^3^, ^4^, ^5^, ^6^

Clinicopathological correlation is necessary for the diagnosis, so biopsy is essential. When SSS is suspected, it is recommended to perform a representative biopsy sample, including skin and muscle fascia; to achieve that, punch biopsies are usually inappropriate. Incisional biopsy under anesthesia at the surgical theater are more likely to obtain an adequate specimen.

Drug therapeutic options are scarce and must be based on antifibrotic properties and the ability to reduce transforming factor β (TGF-β) signaling. Although glucocorticoids inhibit collagen biosynthesis, they are not an appropriate therapeutic option on a continuous basis, due to their side effects. Furthermore, as it is a pauci-inflammatory condition, immunosuppressive and anti-inflammatory medications have not been shown to be effective.^1^

In 2020, a case of reduced disease progression was described with the use of secukinumab, due to its action in reducing TGF-β signaling, a cytokine involved in the pathogenesis of SSS. However, the treatment basis continues to be physical rehabilitation, preferably in the initial stage of the disease.^2^, ^8^, ^10^ Instructing patients to perform motor physical therapy and physical activity is essential.

It is important to recognize SSS as a differential diagnosis of sclerotic diseases, thus preventing ineffective systemic treatment and its possible adverse effects. The detection of histopathological changes requires careful analysis by an experienced dermatopathologist. Studies with a larger sample size and long-term clinical follow-up are required to establish effective therapeutic guidelines.

## Financial support

None declared.

## Authors’ contributions

Jessica Lana Conceição and Silva Baka: Design and planning of the study; collection, analysis and interpretation of data; drafting and editing of the manuscript; critical review of the literature; critical review of the manuscript; approval of the final version of the manuscript.

Tauana Ogata Coelho da Rocha: Analysis and interpretation of data; drafting and editing of the manuscript; critical review of the literature; critical review of the manuscript; approval of the final version of the manuscript.

Marcella Soares Pincelli: Analysis and interpretation of data; drafting and editing of the manuscript; critical review of the literature; critical review of the manuscript; approval of the final version of the manuscript.

Luciana Paula Samorano: Analysis and interpretation of data; drafting and editing of the manuscript; critical review of the literature; critical review of the manuscript; approval of the final version of the manuscript.

Maria Cecília da Matta Rivitti-Machado: Analysis and interpretation of data; drafting and editing of the manuscript; critical review of the literature; critical review of the manuscript; approval of the final version of the manuscript.

Zilda Najjar Prado de Oliveira: Analysis and interpretation of data; drafting and editing of the manuscript; critical review of the literature; critical review of the manuscript; approval of the final version of the manuscript.

## Conflicts of interest

None declared.
